# Comparison of Machine Learning Algorithms for Structure State Prediction in Operational Load Monitoring

**DOI:** 10.3390/s20247087

**Published:** 2020-12-10

**Authors:** Waldemar Mucha

**Affiliations:** Department of Computational Mechanics and Engineering, Silesian University of Technology, 44-100 Gliwice, Poland; waldemar.mucha@polsl.pl; Tel.: +48-32-237-12-04

**Keywords:** operational load monitoring, ANFIS, support-vector machine, gaussian process regression, structural health monitoring, strain measurement, hat-stiffened panel

## Abstract

The aim of operational load monitoring is to make predictions about the remaining usability time of structures, which is extremely useful in aerospace industry where in-service life of aircraft structural components can be maximized, taking into account safety. In order to make such predictions, strain sensors are mounted to the structure, from which data are acquired during operational time. This allows to determine how many load cycles has the structure withstood so far. Continuous monitoring of the strain distribution of the whole structure can be complicated due to vicissitude nature of the loads. Sensors should be mounted in places where stress and strain accumulations occur, and due to experiencing variable loads, the number of required sensors may be high. In this work, different machine learning and artificial intelligence algorithms are implemented to predict the current safety factor of the structure in its most stressed point, based on relatively low number of strain measurements. Adaptive neuro-fuzzy inference systems (ANFIS), support-vector machines (SVM) and Gaussian processes for machine learning (GPML) are trained with simulation data, and their effectiveness is measured using data obtained from experiments. The proposed methods are compared to the earlier work where artificial neural networks (ANN) were proven to be efficiently used for reduction of the number of sensors in operational load monitoring processes. A numerical comparison of accuracy and computational time (taking into account possible real-time applications) between all considered methods is provided.

## 1. Introduction

Operational load monitoring (OLM) is a process that consists in investigating characteristics of a structure under mechanical loads in its operating environment, be means of acquiring and saving data from sensors mounted to the structure. The gathered data are then processed to estimate the remaining in-service life of the structure, taking into account the number and characteristics of load cycles that the structure has withstood so far [1,2,3]. OLM processes are mainly utilized in aerospace industry for monitoring aircraft structures in order to maximize their in-service life while ensuring flight safety [4,5]. Such approach reduces the costs of inspections and increases the aircraft availability [6].

A related process to operational load monitoring, is Structural Health Monitoring (SHM), where the mechanical system is monitored, often in real time, using sensor data, in order to detect potential reduction of system performance, or even damage [7,8,9]. In aerospace industry, often the both processes are performed simultaneously (Structural Health and Usage Monitoring Systems, HUMS [1,10]), where the strains and stresses at given points are measured, and a real-time system combines the data with flight parameters to determine the current state of the structure; the data are then utilized to compute fatigue factors of some components [11]. Sometimes, OLM is considered as a case of SHM [2].

Typical sensors utilized for OLM are embedded (intrinsic optical fiber) and surface mounted (strain gauges) strain sensors [12,13]. These sensors should be placed in critical points (places of expected stress and strain accumulations). Due to vicissitude nature of the loads, and therefore variable distributions of strains and stresses during operations, the number of required sensors may be high. As the accuracy of OLM depends on the number of sensors, and the number is limited, monitoring of flying parameters (like speed, acceleration and mass) is often performed to estimate the state of the structure in other locations [14]. In the author’s earlier work [15] it has been proven that by implementing artificial neural networks (ANNs), the temporary state of the whole structure could be quite accurately estimated using only a relatively small number of sensors. The neural network was trained with data obtained from finite element simulations of the structure under many different load cases, and then tested with data acquired from strain gauges in series of experiments.

The following work is the analysis of implementation possibilities of other machine learning and artificial intelligence algorithms to OLM, for estimation of the state of the monitored structure based on a reduced number of strain sensor reads. Three different techniques are considered: adaptive neuro-fuzzy inference systems (ANFIS), support-vector machines (SVM) and Gaussian processes for machine learning (GPML). Each of these techniques was used to build several models with different parameters, that were trained by simulation data. All of the models were tested with experimental data, the accuracy and computational time was measured (taking into account possible real-time OLM applications). Detailed comparison between the obtained results is provided.

As there is a potential of real-time applications of machine learning or artificial intelligence methods implemented to OLM processes, the possibilities to deploy such models to real-time systems or embedded systems are considered. Regarding ANNs, the most popular approach is to implement already trained networks (on a capable platform, like PC) to the embedded device. As ANNs are algorithms of relatively low complexity, a whole variety of such applications can be found in the literature, regarding classification of signals for arrythmia detection [16], evaluation of fiber–metal laminates [17], Secchi depth calculation [18], power factor correction [19], or hybrid simulation [20]. Implementing SVM algorithms to real-time systems or embedded systems is also a popular approach. Zhang et al. [21] implemented SVM to detect ventricular fibrillation in real time, Arsalane et al. [22] deployed SVM to an embedded system for meat freshness evaluation based on machine vision. Dey et al. [23] studied performance of SVM classification algorithms running on embedded processors and suggested parallel computations. There are also examples of SVMs implemented to FPGA-based systems, like speech recognition system [24] or melanoma detection [25]. Regarding ANFIS, there are many examples of real-time applications of these algorithms in automatic control area, like of hydraulic systems [26], active suspension system for passenger comfort improvement [27], robot arms [28], or mobile robots navigation for target seeking and obstacle avoidance [29]. Karakuzu et al. [30] and Maldonado et al. [31] deployed ANFIS algorithms to FPGAs. Danzomo et al. [32] implemented an ANFIS-based algorithm to Arduino microcontroller, Sakib et al. [33] to Raspberry Pi for flood monitoring system, and Mishra et al. [34] to a real-time system with DSP 2812. Although real-time applications of GPML models are known [35,36], examples of deploying these algorithms to embedded devices were not found, which is probably caused by the computational complexity of these models (they require all the training data to make predictions).

## 2. Materials and Methods

**Figure 1:**
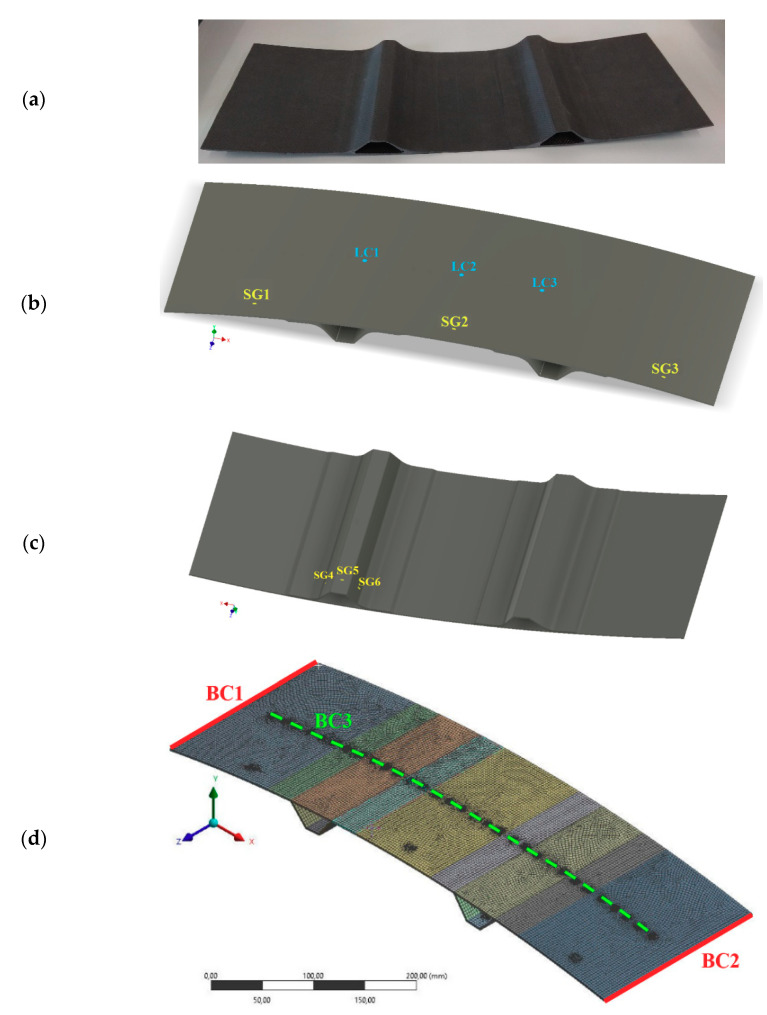
Considered aerostructure: (**a**) photograph, (**b**) top strain gauges SG1–SG3 positions and load points LC1–LC3, (**c**) bottom strain gauges SG4–SG6 positions, (**d**) finite element model and boundary conditions. Reprinted from [15].

A typical structure for aerospace applications is considered—hat-stiffened composite panel made of carbon/epoxy woven laminate, presented in Figure 1a. In the author’s earlier work [15], a detailed description of the geometry, experimental measurements of the mechanical properties, as well as building and validating a finite element model, can be found. 

Six strain gauges (SG1–SG6) are mounted to the panel, in positions presented in Figure 1b,c. Strain measurements are performed in longitudinal directions. Finite element model of the panel is presented in Figure 1d. It consists of 26,357 surface elements and 79,217 nodes. At the edge, BC1 displacements in directions X and Y are fixed, at the opposite edge (marked as BC2) displacements in direction Y are fixed. The panel is loaded vertically at any point on the line BC3 (the load is applied to a 5 mm circle surface, displacements along Z axis are fixed within the circle).

**Figure 2:**
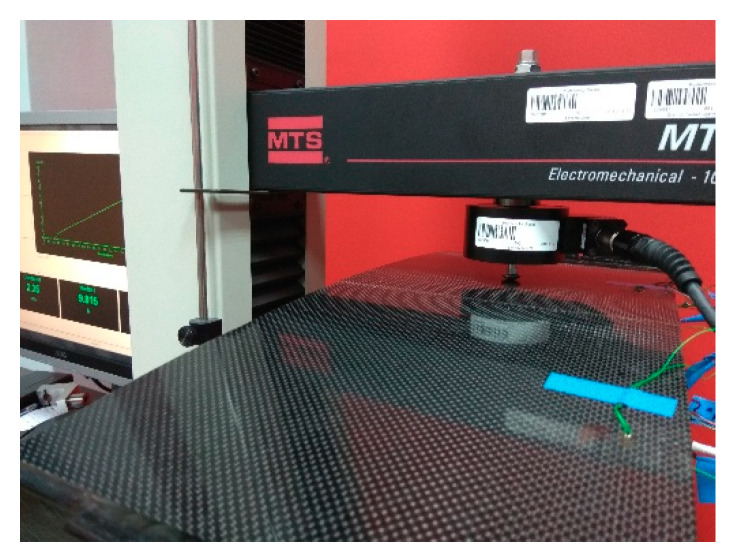
Experimental testing. Reprinted from [15].

The experimental tests were performed using universal testing machine MTS Insight 10 with 500 N load cell (MTS Systems Corporation, Eden Prairie, MS, USA) (Figure 2). The panel was mounted in a specially designed stiff frame (to mimic the boundary conditions BC1–BC3) and loaded in points LC1–LC3, of location presented in Figure 1b. Data from strain gauges and load cell were acquired using HBM AB22A hardware system (Hottinger Baldwin Messtechnik, Darmstadt, Germany) and Catman Easy software. The force signal from the load cell was received at the analog input of the acquisition system. Data from strain gauges and load cell were acquired with 20 Hz sampling frequency. No preprocessing was applied to the measured signals.

For OLM purposes, maximum inverse safety factor of the whole structure was observed during loading with different force values, and at different points. Safety factor is usually defined as the margin to failure, the inverse safety factor ISF_C*i*_ is denoted as the quotient of the applied load *Q* to the failure load *Q*_C*i*_, taking into account a given failure criterion C*i*:

(1) $$ISF_{Ci}=\frac{Q}{Q_{Ci}}.$$

when there are *n* failure criteria (i=1,2,…,n), the global inverse safety factor ISF is calculated as the maximum value from each criteria:

(2) $$ISF=\max(ISF_{Ci})$$

In the presented research, three failure criteria were considered: maximum stress criterion C_1_, maximum strain criterion C_2_ and Tsai-Wu [37] criterion C_3_:

(3) $$ISF_{C1}=\max\left(\frac{\sigma_{j}}{\sigma_{\lim,j}}\right),$$

(4) $$ISF_{C2}=\max\left(\frac{\varepsilon_{j}}{\varepsilon_{\lim,j}}\right),$$

(5) $$ISF_{C3}=F_{j}\;\sigma_{j}\;+\;F_{jk}\;\sigma_{j}\sigma_{k},$$

where *j,k* = 1,2,…,6, *σ_j_* and *ε_j_* are stresses and strains in Voight notation, respectively, *F_i_* are parameters calculated from stress limits *σ*_lim,*j*_, and *F_jk_* are coupling coefficients. In criteria C_1_ and C_2_, different stress *σ*_lim,*j*_ and strain *ε*_lim,*j*_ limits were taken for tension and compression.

**Figure 3:**
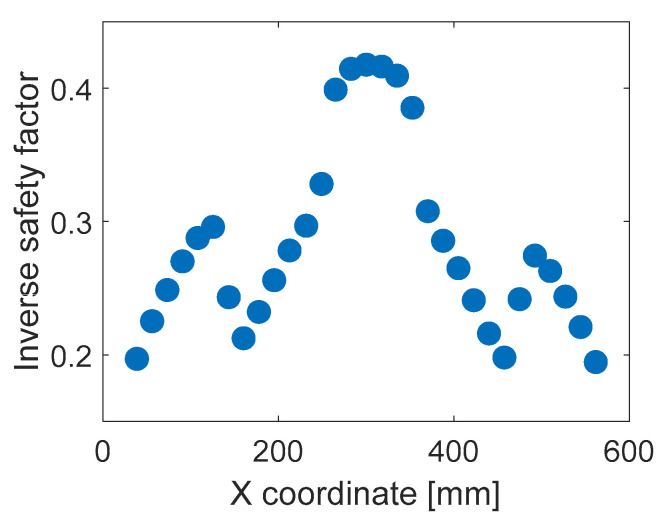
Generated data samples of ISF (inverse safety factor) for example force value of 40 N.

From the finite element (FE) model, 1241 data samples of relationship between strains in positions SG1-SG6 and the value of *ISF* (Supplementary S7 to [15]) were generated. When generating the data, forces of different values were applied to 31 spots at line BC3 (Figure 1d). Figure 3 illustrates the nonlinear behavior of the *ISF*, where it is plotted against location of the applied force (all generated data samples with the same example force value of 40 N).

**Figure 4:**
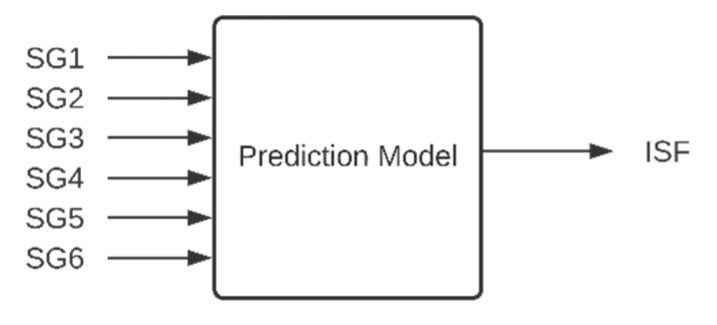
Inputs and outputs of the prediction model.

The authors of [15] present a neural network, trained with 70% of the abovementioned data generated from FE simulations, that predicts the maximal value of *ISF* for the whole structure based on the data from strain gauges (Figure 4). Efficiency of the networks, when dealing with strain values acquired in series of experiments, was measured.

The following work presents comparison between results of the ANN presented in [15] and of implementation of three other machine learning methods (similar prediction models, of inputs and output as presented in Figure 4): ANFIS, SVM, and GPR. The comparison takes into account the accuracy of the results and the computational effort for making predictions. In the presented research, white gaussian noise was added to the input data (SG1–SG6) generated from FE simulations (Supplementary Materials S1) in order to build more robust models to noisy data.

**Figure 5:**
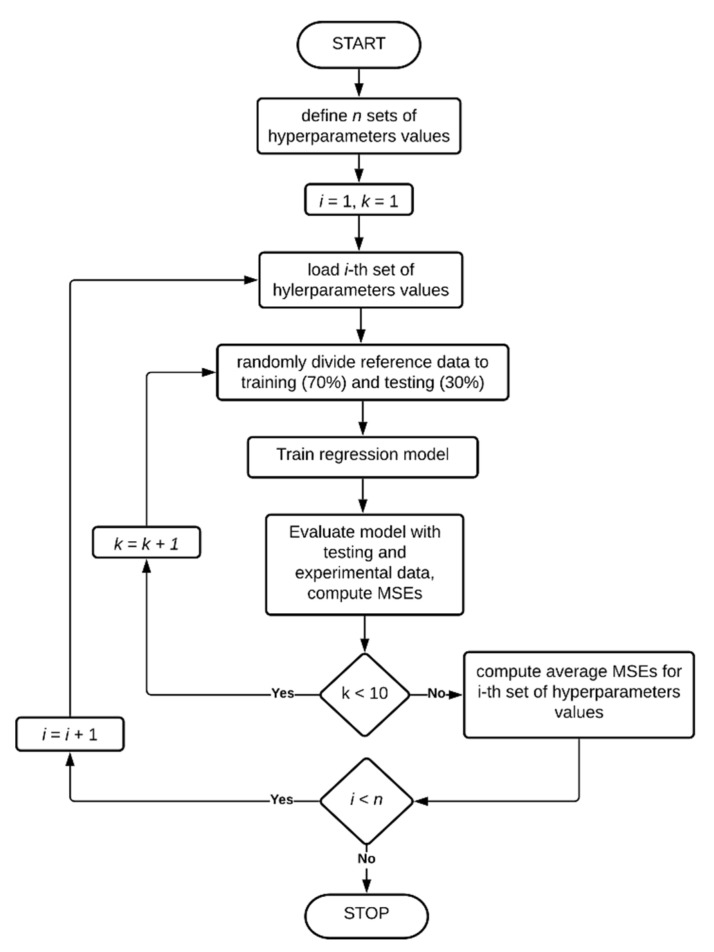
Algorithm for finding best hyperparameters values for each model.

Before building the models, a grid search approach was implemented to find the best values of hyperparameters of the models, according to the algorithm presented in Figure 5. For each set of hyperparameters values, the model was trained 10 times, each time with randomly chosen 70% of the reference data. Each time the model was tested with remaining 30% of the reference data and with experimental data. Mean squared errors (MSEs) were computed. Each set of hyperparameters values was evaluated by the average MSE from 10 iterations.

**Figure 6:**
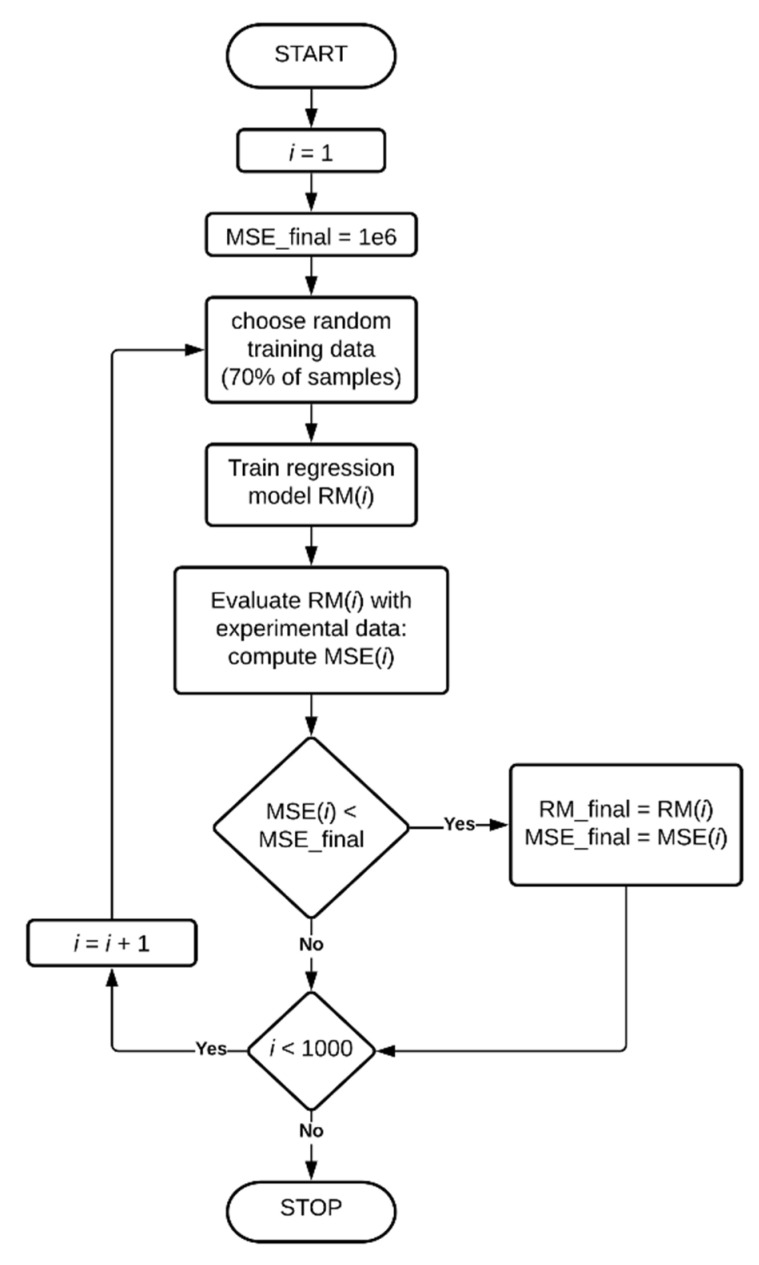
Algorithm for finding best suited models of each type.

After choosing the best combination of hyperparameters values, the models were build using algorithm presented in Figure 6. One thousand attempts for training the regression model were performed, from which the one that gave the least mean squared error (MSE) of ISF, when introduced to input data acquired from series of experiments, compared to reference output obtained from FE simulations (knowing the applied force), was chosen.

The algorithms presented in Figure 5 and Figure 6 were applied to ANFIS, SVM and GPR models.

**Figure 7:**
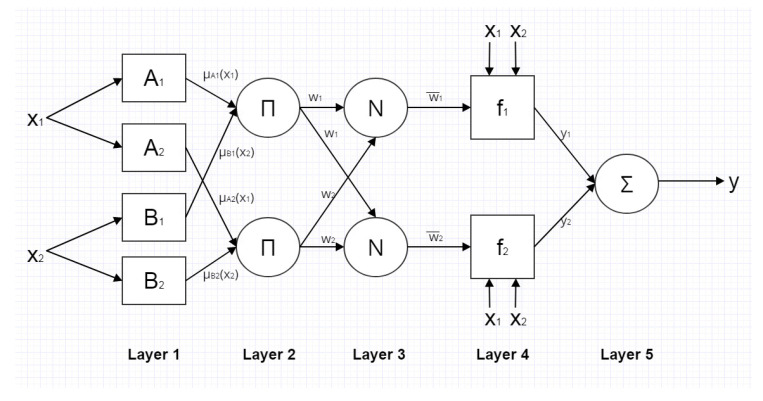
Structure of ANFIS.

ANFIS is a fuzzy inference system implemented in the framework of adaptive networks [38]. It has the possibility to integrate the benefits of both fuzzy inference systems and adaptive neural networks. In fuzzy systems the output is mapped from the input, based on fuzzy logic (involving membership functions, fuzzy logic operators and if-then rules). Artificial neural networks, on the other hand, are collections of artificial neurons, a mathematical model of biological neurons. The artificial neurons (nodes) are connected with directional links to transmit signals. If some of the nodes’ outputs depend on a set of parameters (to minimize the error by changing the parameters during learning), than it is an adaptive neural network [39,40]. The ANFIS structure consists of five layers, as presented in Figure 7 of network with two inputs *x*_1_ and *x*_2_.

Layer 1 is the fuzzification layer, where membership degrees of the inputs to rules are computed using the membership functions (MFs) *μ*(*x_i_*). In the second layer (the rule layer), the firing strengths of each rule is determined as the product of inputs:

(6) $$w_{i}=\mu_{Ai}\cdot \mu_{Bi},$$

for *i* = 1, 2. In the third layer, the input values are normalized as follows:

(7) $$\bar{w_{i}}=\frac{w_{i}}{w_{1}+w_{2}},$$

for *i* = 1, 2. The fourth layer is known as the defuzzification layer with adaptive nodes that perform the following function:

(8) $$y_{i}=\bar{w_{i}}\left(p_{i}x_{1}+q_{i}x_{2}+r_{i}\right),$$

where *p_i_*, *q_i_* and *r_i_* are consequent parameters for rule *i* = 1, 2. The last, fifth layer is the output layer that calculates the sum of its inputs. 

ANFIS can learn from training data the same way as ANNs; however, the advantage of ANFIS over ANN is that the hidden layer in ANFIS is already determined by the fuzzy interference system, and its size does not have to be found. ANFIS techniques have been implemented for prediction tasks in many different areas of engineering applications, like biomechanics [41], geotechnics [42], production technology [43], failure analysis [44] or structural mechanics [45].

When finding the most suited ANFIS for the given problem, algorithms presented in Figure 5 and Figure 6 were implemented to MATLAB (utilizing Fuzzy Logic Toolbox [46]). The following membership functions *μ*(*x_i_*) types were considered: generalized bell-shaped (9), gaussian (10), gaussian combination (where left and right curves are of type (10)), triangular (11), trapezoidal (12), difference between two sigmoidal functions (where a single function is given as (13)), product of two sigmoidal functions (where a single function is given by (13)), Pi-shaped (14):

(9) $$\mu \left(x_{i},a,b,c\right)=\frac{1}{1+{\left|\frac{x_{i}-c}{a}\right|}^{2b}},$$

(10) $$\mu \left(x_{i},a,b\right)=\exp\left(\frac{-{\left(x_{i}-b\right)}^{2}}{2a^{2}}\right),$$

(11) $$\mu \left(x_{i},a,b,c\right)=\max\left(\min\left(\frac{x_{i}-a}{b-a},\;\frac{c-x_{i}}{c-b}\right),0\right),$$

(12) $$\mu \left(x_{i},a,b,c,d\right)=\max\left(\min\left(\frac{x_{i}-a}{b-a},1,\;\frac{d-x_{i}}{d-c}\right),0\right),$$

(13) $$\mu \left(x_{i},a,b\right)=\frac{1}{1+e^{-a\left(x_{i}-c\right)}},$$

(14) $$\mu \left(x_{i},a,b,c,d\right)=\{\begin{array}{cc} 0,\; & x_{i}\leq a\\ 2{\left(\frac{x_{i}-a}{b-a}\right)}^{2}, & a\leq x_{i}\leq \frac{a+b}{2}\\ 1-2{\left(\frac{x_{i}-b}{b-a}\right)}^{2}, & \frac{a+b}{2}\leq x_{i}\leq b\\ 1, & b\leq x_{i}\leq c\\ 1-2{\left(\frac{x_{i}-c}{d-c}\right)}^{2}, & c\leq x_{i}\leq \frac{c+d}{2}\\ 2{\left(\frac{x_{i}-d}{d-c}\right)}^{2}, & \frac{c+d}{2}\leq x_{i}\leq d\\ 0, & x_{i}\geq d \end{array},$$

where *a*, *b*, *c*, *d* are parameters to fit. The type of membership functions was the hyperparameter to be optimized with algorithm from Figure 5.

Among machine learning tools, support vector machines (SVMs) are supervised learning algorithms used in classification and regression problems. SVMs are based on statistical learning theory proposed by Vapnik [47] where training data are represented as points in space divided by a gap. The points are mapped to maximize the gap size. When data for classification is introduced, it is mapped in the same space and classified based on location of each point. In order to use SVMs for nonlinear classification, kernel trick is implemented, where inputs are mapped to higher-dimensional space [48]. Points in the new space are defined by a kernel function *k*(*x*, *y*). A hyperplane is found, for which the distance to nearest data points (defined as margin) on both sides is as large as possible. The hyperplane is defined as:

(15) wx−b=0,

where *w* is the normal vector to the hyperplane and *b* is constant. In SVMs used for regression, the model depends only on a subset of the training data limited by parameter *ε* (which defines how much error is acceptable in the model). When training a regression SVM [49], the following minimization is performed:

(16) $$\frac{1}{2}{\left\|w\right\|}^{2}\to \min$$

where

(17) $$\left|y_{i}-\langle w,x_{i}\rangle -b\right|\leq \varepsilon .$$

The weight vector *w* can be presented as the linear combination of the training data:

(18) $$w=\sum_{i=1}^{N}\left(\alpha_{i}-\alpha_{i}{}^{*}\right)x_{i},$$

*α_i_* and α_i_^*^ being the coefficients associated with training points. The regression function takes the form:

(19) $$f\left(x\right)=\sum_{i=1}^{N}\left(\alpha_{i}-\alpha_{i}{}^{*}\right)k\left(x_{i},x\right)+b.$$

It has been proven that SVMs are effective regression tools for nonlinear data [50,51,52,53]; therefore, it is motivating to measure the effectiveness of SVMs used for structure state predictions in OLM.

The computations were performed using MATLAB and Statistics and Machine Learning Toolbox [54]. Three different kernel functions were considered: gaussian

(20) $$k\left(x_{i},x\right)=\exp\left(-{\left\|x_{i}-x\right\|}^{2}\right),$$

linear

(21) $$k\left(x_{i},x\right)=x_{i}{}^{T}x,$$

and polynomial

(22) $$k\left(x_{i},x\right)={\left(1+x_{i}{}^{T}x\right)}^{q}.$$

The optimized hyperparameters were *ε* (half of the width of the epsilon-insensitive band) for kernels (20)–(22), kernel scale for (20), and polynomial order *q* for kernel function (22). 

Constraint *C* for coefficients *α_i_* and α_i_^*^

(23) $$\forall i:0\leq \alpha_{i},\alpha_{i}{}^{*}\leq C$$

was calculated as a quotient of the interquartile range of the target vector *y*:

(24) $$C=\frac{\mathrm{iqr}\left(y\right)}{1.349}.$$

The utilized optimization routine was Sequential Minimal Optimization.

The third considered technique is Gaussian Process Regression (GPR), a GPML method [55]. The process is based on Bayesian framework where predictive output is provided based on prior conditional probability. The key assumption is that the probability distribution for any input vector *x* ∈ **R** (and target values *y* ∈ **R**) over the target function *y* = *f*(*x*) follows the Gaussian distribution:

(25) $$f\left(x\right)∼N\left(\mu \left(x\right),K\left(x,x_{i}\right)\right)$$

where *μ*(*x*) is the associated mean function and *K*(*x*, *x_i_*) is the associated covariance (kernel) function [56,57]. The model parameters are chosen in an optimization process. Output value of an unseen point is predicted from the similarity between points *K* [58]. The main disadvantage of GPR is that it uses the whole training data for predictions; however, it was proven to be an efficient tool for making predictions [57,58,59], also when dealing with nonlinearities and uncertainties between inputs and targets [60,61,62]. Moreover, it was reported that GPR processing often requires less input data than ANNs [63,64,65]. 

Four GPR models were found using the algorithms presented in Figure 5 and Figure 6. Different covariance functions were introduced to each of the model:

(26) $$K\left(x,x_{i},\sigma_{f},\sigma_{l}\right)=\sigma_{f}{}^{2}\exp\left(-\frac{1}{2}\frac{{\left(x-x_{i}\right)}^{T}\left(x-x_{i}\right)}{\sigma_{l}{}^{2}}\right),$$

(27) $$K\left(x,x_{i},\sigma_{f},\sigma_{l}\right)=\sigma_{f}{}^{2}\exp\left(-\frac{\sqrt{{\left(x-x_{i}\right)}^{T}\left(x-x_{i}\right)}}{\sigma_{l}}\right),$$

(28) $$K\left(x,x_{i},\sigma_{f},\sigma_{l}\right)=\sigma_{f}{}^{2}\left(1+\frac{\sqrt{5{\left(x-x_{i}\right)}^{T}\left(x-x_{i}\right)}}{\sigma_{l}}+\frac{5{\left(x-x_{i}\right)}^{T}\left(x-x_{i}\right)}{3\sigma_{l}{}^{2}}\right)exp\left(\frac{-\sqrt{5{\left(x-x_{i}\right)}^{T}\left(x-x_{i}\right)}}{\sigma_{l}}\right),$$

(29) $$K\left(x,x_{i},\sigma_{f},\sigma_{l},\alpha \right)=\sigma_{f}{}^{2}{\left(1+\frac{{\left(x-x_{i}\right)}^{T}\left(x-x_{i}\right)}{2\alpha \sigma_{l}{}^{2}}\right)}^{-\alpha },$$

where *σ_f_*, *σ_l_* and *α* are the parameters of the functions [66]. Equation (26) represents the squared exponential covariance function (implemented in model 1), (27) the exponential function (model 2), (28) the Matern 5/2 function (model 3), and (29) the rational quadratic function (model 4). The optimized hyperparameter for each model was the initial value for the noise standard deviation *σ*_0_.

## 3. Results

All considered regression models are listed in Table 1, each of these models were given a short designation. Grid-search optimization was performed for all the models using algorithm presented in Figure 5. When the best hyperparameter values were revealed, the models were built using algorithm from Figure 6. Finally, all the obtained models were tested using both numerical and experimental input data, their prediction accuracy and time of computations were measured in order to provide detailed comparison.

### 3.1. ANFIS Models

Two ANFIS models were considered: with two membership functions per each input (64 rules) and with three membership functions per each input (729 rules). Algorithm presented in Figure 5 was applied, do determine the best type of membership function. In the result of the grid search, for both ANF1 and ANF2 models, the most accurate regression models are with triangular membership functions. 

**Figure 8:**
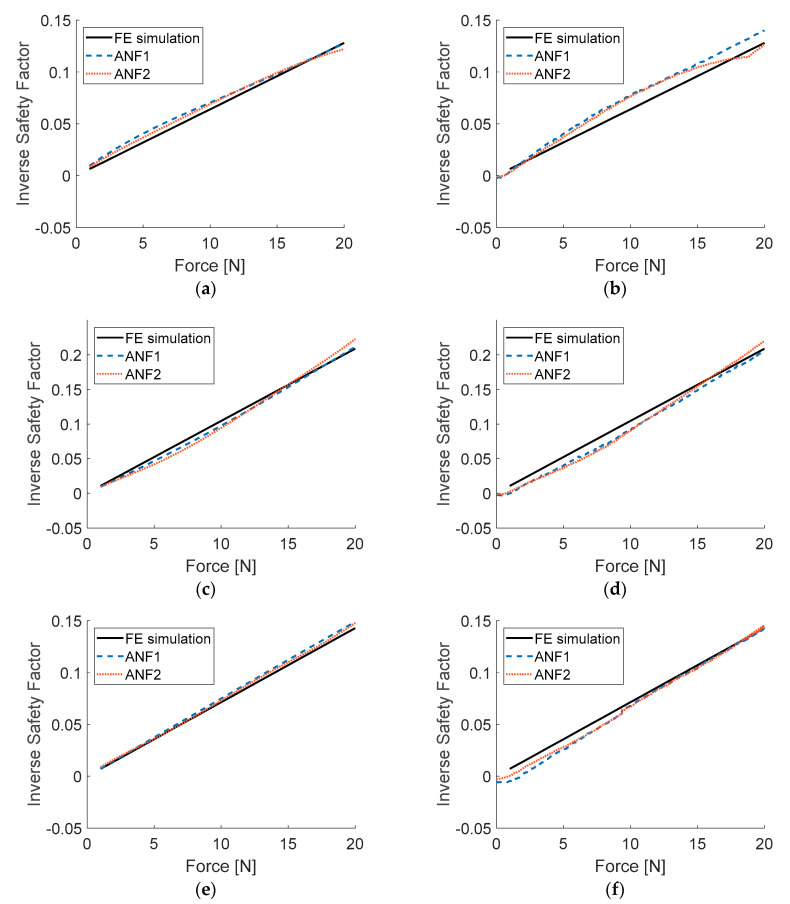
Results of testing ANFIS models: (**a**) load case LC1, numerical input data; (**b**) load case LC1, experimental input data; (**c**) load case LC2, numerical input data; (**d**) load case LC2, experimental input data; (**e**) load case LC3, numerical input data; (**f**) load case LC3, experimental input data.

Figure 8 presents the efficiency of the models trained according to algorithm presented in Figure 6. Results of ISF when introduced to input data from numerical simulations, as well as from series of experiments, were compared to reference data (obtained from FE simulations, for experimental inputs based on measured values of applied loads).

Table 2 presents the comparison of accuracy and required computational effort for the obtained models. The accuracy is measured as the root mean square error (RMSE) between the model output and the reference ISF value obtained from numerical simulations. The required computational effort is measured as the total time of computations of every point in the experimental input dataset (1308 points). The computational time was measured as the average time from 100 runs in MATLAB software, on a PC (AMD FX-6350 processor, 16 GB DDR3 RAM).

RMSE obtained from testing with data acquired during experiments are considered to be much more relevant than RSME concerning numerically generated input data. As presented in Table 2, ANF2 model requires over 10 times more computational effort than ANF1 model, while returning results of about 12% smaller RMSE. However, as one can see in Figure 8, the results obtained from ANF1 and ANF2 models do not differ substantially (both RSME values are relatively small) therefore, ANF1 model will be taken for further considerations. Possible real-time applications of ANF2 model raises doubts for its high computational complexity with only a small improvement of accuracy, compared to ANF1.

### 3.2. SVM Models

Three SVM models were considered (Table 1). The following hyperparameters were optimized using the grid-search approach: kernel scale *s* and half of the width of the epsilon-insensitive band *ε* for SVM1 (gaussian) model, only *ε* for SVM2 (linear) model, polynomial order *q* and *ε* for SVM3 (polynomial) model. The following ranges of the hyperparameters were considered:

(30) $$s\in \left[10^{-3},10^{3}\right],$$

(31) $$\varepsilon \in \left[10^{-3},10^{2}\right]\cdot \frac{\mathrm{iqr}\left(y\right)}{1.349},$$

(32) q∈{2,3,4}.

For *s* and *ε* 50 logarithmically equally spaced points in the ranges (30) and (31) were created. For SVM1 model, the following values were obtained: *s* = 1 and *ε* = 0.0178·iqr(*y*)/1.349. For both SVM2 and SVM3 models, the optimal *ε* value was 0.3162, and the optimal polynomial order for SVM3 model was 2.

**Figure 9:**
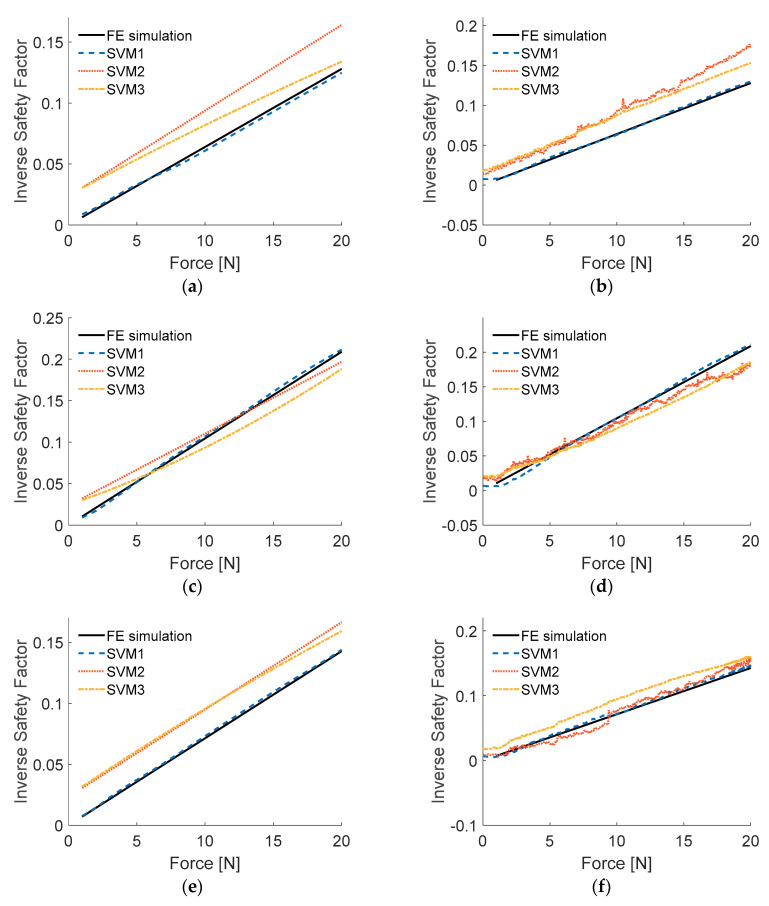
Results of testing SVM models: (**a**) load case LC1, numerical input data; (**b**) load case LC1, experimental input data; (**c**) load case LC2, numerical input data; (**d**) load case LC2, experimental input data; (**e**) load case LC3, numerical input data; (**f**) load case LC3, experimental input data.

The three SVM models with optimal values of hyperparameters were obtained with algorithm presented in Figure 6 (each one is the best from 1000 trials). The models were tested with numerical and experimental input data similarly as ANFIS models, the output plots are presented in Figure 9.

**Figure 10:**
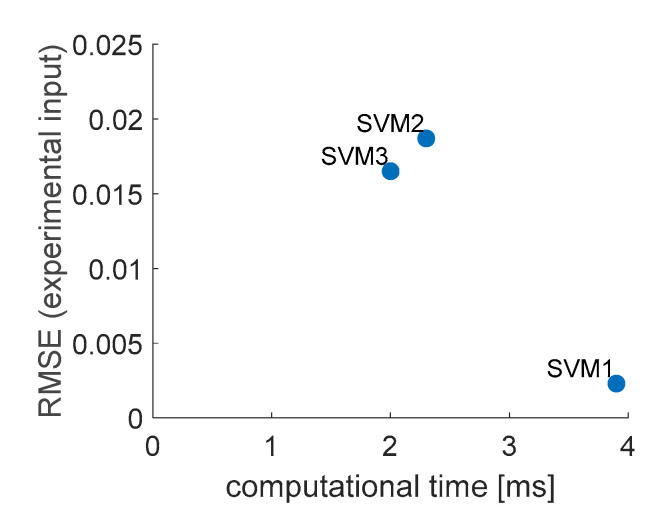
Graphical comparison of accuracy and computational time for obtained SVM models.

The accuracy and required computational effort were measured the same way as with ANFIS models. The comparison is presented in Table 3, and graphically (simplifying the accuracy to experimental input data) in Figure 10. Evident conclusion from Figure 10 is that model SVM2 should be ignored in further considerations, as SVM3 performs better in both considered criteria. As one can see in Figure 9, the linear model returned quite noisy results when dealing with experimentally measured inputs, although it was trained on data with white gauss noise added. The gaussian model proved to be most immune to noisy experimental input data. SVM1 will be taken for further considerations, as it is characterized by RMSE nearly seven times smaller than SVM3 and while the computational time of both models is of the same order of magnitude.

### 3.3. GPR Models

For four GPR models (according to Table 1) with different covariance functions, grid search of one hyperparameter was performed—initial value for the noise standard deviation *σ*_0_ in the range [10^−4^, 1], where 50 logarithmically equally spaced points were created. The following optimal values were revealed: 0.0681, 0.1468, 0.0015, 0.4642 for models GPR1–GPR4, respectively.

**Figure 11:**
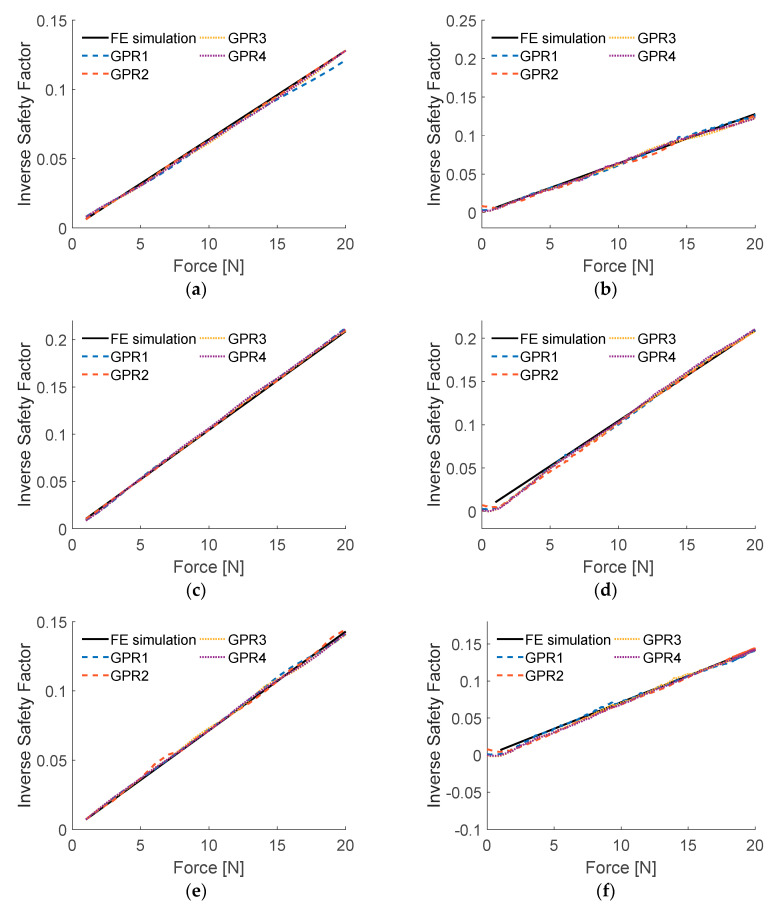
Results of testing GPR models: (**a**) load case LC1, numerical input data; (**b**) load case LC1, experimental input data; (**c**) load case LC2, numerical input data; (**d**) load case LC2, experimental input data; (**e**) load case LC3, numerical input data; (**f**) load case LC3, experimental input data.

**Figure 12:**
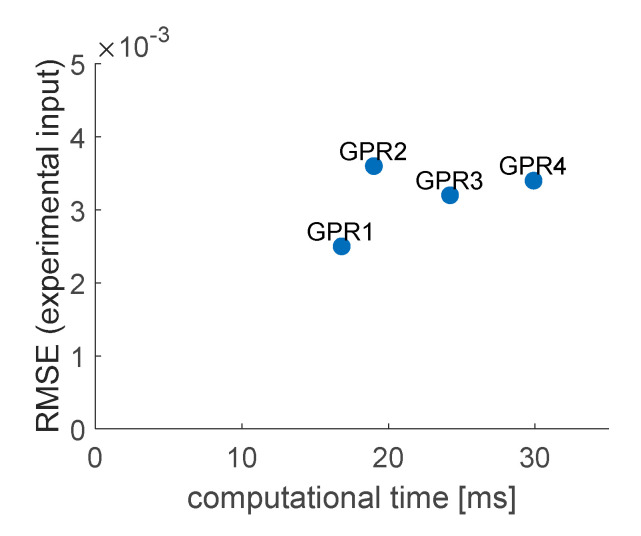
Graphical comparison of accuracy and computational time for obtained GPR models.

Taking into account the found best hyperparameter values, final models GPR1–GPR4 were created with algorithm presented in Figure 6. Figure 11 contains the IRF plots of the obtained models, obtained for numerical and experimental input data, as previously. Table 4 compares the accuracy and required computational effort for GPR models, measured the same way as ANFIS and SVM models. The same comparison was graphically illustrated in Figure 12, for better visibility.

When dealing with numerical input data, the results of GPR1, GPR3 and GPR4 models are of similar accuracy, GPR2 is a little more accurate. However, when introduced to data acquired from experiments, GPR1 model turned out to be most efficient, returning results 20–30% more accurate than other models and requiring the least computational time from all GPR models. Although the accuracy of all the obtained models is satisfying, it can be clearly seen in Figure 12 that GPR1 model outruns GPR2–GPR4 models in both considered criteria; therefore, GPR2–GPR4 (of very similar accuracy when introduced to experimentally acquired input data) will be excluded from further considerations.

### 3.4. Comparison of ANFIS, SVM, GPL and ANN Models

The chosen models of ANFIS, SVM and GPR methods were compared to each other and to the artificial neural network from the previous research [15]. There were 100,000 trials for training the ANN, each time with a random number of neurons in the hidden layer, between 3 and 20. The ANN with be smallest error when dealing with experimental input, was chosen as the final one. The final ANN had nine neurons in the hidden layer.

**Figure 13:**
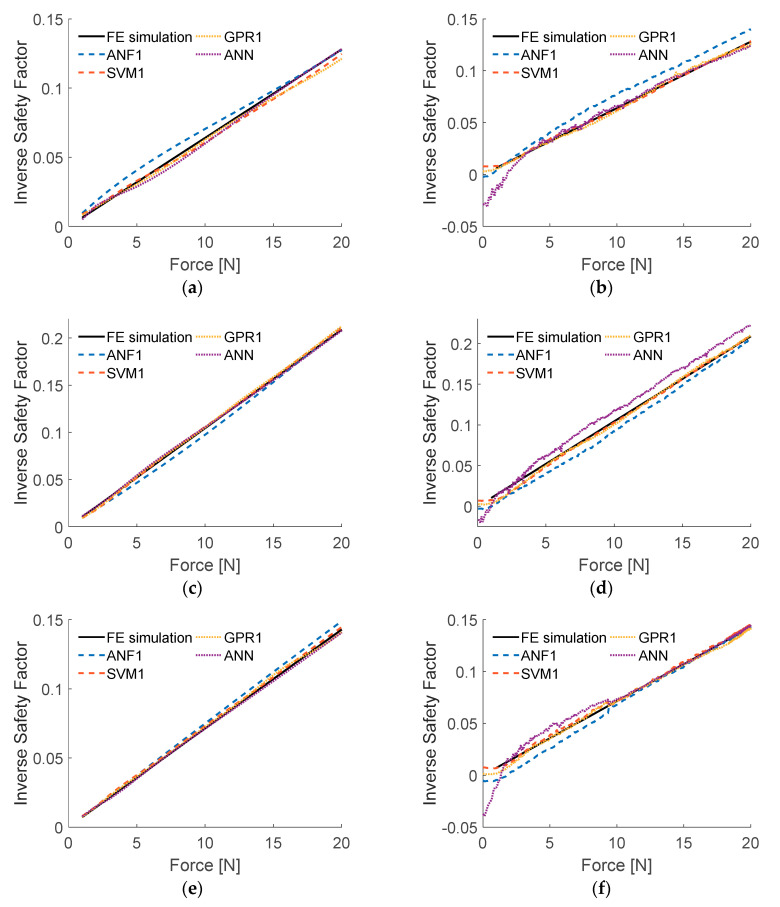
Results of testing chosen models of different methods: (**a**) load case LC1, numerical input data; (**b**) load case LC1, experimental input data; (**c**) load case LC2, numerical input data; (**d**) load case LC2, experimental input data; (**e**) load case LC3, numerical input data; (**f**) load case LC3, experimental input data.

**Figure 14:**
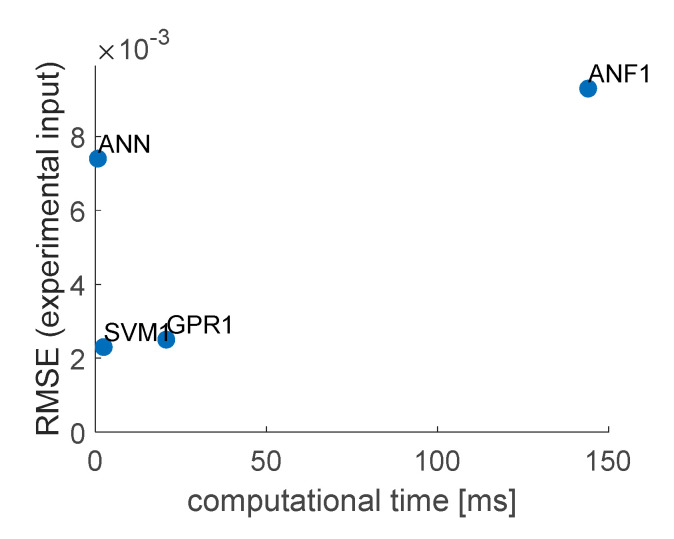
Graphical comparison of accuracy and computational time for chosen models.

Figure 13 presents the models’ responses to time series of strain data SG1–SG6, generated numerically and measured experimentally, for load cases LC1–LC3. Calculated root mean square errors and measured computational times are compared in Table 5. Graphical comparison of accuracy (with experimental input) and computational time of the models is presented in Figure 14. The ANN turned out to be computationally the most efficient and SVM1 the most accurate (about three times more accurate than ANN, but requiring about 3.5 times more computational time). The worst from all compared models was the ANF1, as its accuracy and computational time were far worse than of all the rest. The GPR1 model was of very similar accuracy than SVM1 (8% worse) and about nine times slower.

Statistical significance of the obtained results from different models was also measured with series of *t*-tests. Table 6 and Table 7 list the results of the *t*-tests (*p*-values) when comparing the outputs of different models introduced to numerically computed and experimentally measured input data, respectively. The null hypothesis was that the performance metrics of two considered models are equal. If the significance level was assumed as 5%, when considering numerical input data, the null hypothesis was failed to be rejected for comparing GPR1 and SVM1 models, and when considering experimental input data, the null hypothesis was failed to be rejected for pairs ANF1-SVM1 and ANF1-GPR1.

**Figure 15:**
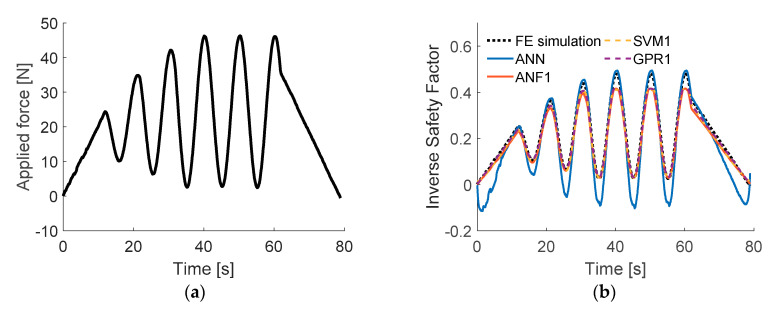
Results of testing chosen models with experimental input from Supplementary S8 of [15]: (**a**) time plot of the applied force; (**b**) time plots of the ISF obtain from different models.

The models were also tested with measured input data during real-time experiment described in [15] (Supplementary Materials S8 to [15]) where the force was applied at point LC2 (the time plot of the force and the obtained results are presented in Figure 15). When the training data were generated, the forces did not exceed the value of 40 N in the simulations. What is curious about these results is that the applied force exceeded that value (the last four peaks), and only the ANN was able to make proper ISF predictions there, the rest of the models stopped at earlier values. The ANN on the other hand did not predict well the low values of ISF (below 0.1) however, such values are often insignificant when the strength of materials is taken into account.

**Figure 16:**
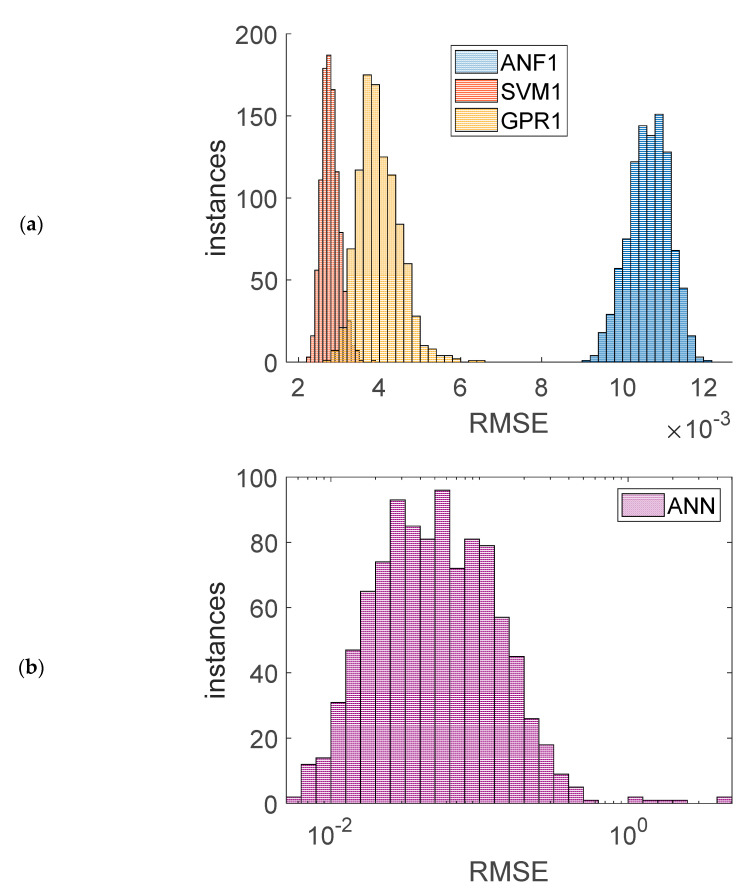
Histograms of RMSE for training 1000 models: (**a**) ANF1, SVM2, and GPR2 models, (**b**) ANN models.

**Figure 17:**
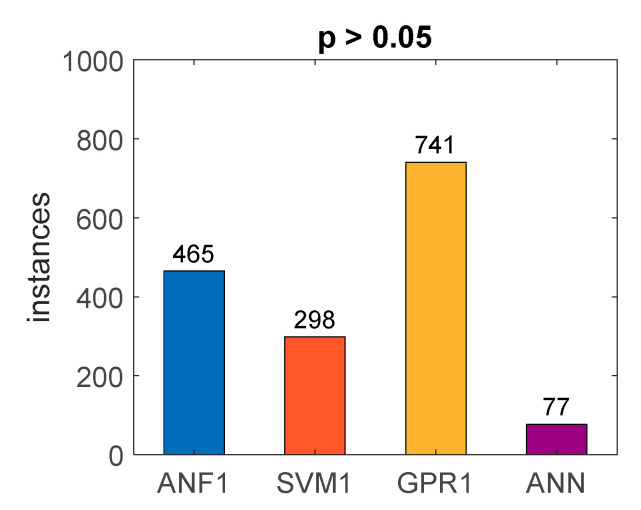
Results of *t*-test repeatability for chosen models.

Finally, a statistical research was performed, regarding repeatability of the accuracy of the models during training trials. RSME of the output, when dealing with noisy experimentally measured input data, was measured in the similar way as listed in Table 2, Table 3, Table 4 and Table 5. For ANF, GPR, and SVM models, 1000 new training trials were started with identical (best) hyperparameters values. For ANN model, 1000 new training trials were started using the same training parameters as described in [15] (nine hidden neurons, and the same training routine). For each trial, accuracy of the model was measured by computing RMSE value, and a *t*-test of pairwise comparison of the trained model and the considered model (found with algorithm from Figure 6) was performed. Histograms of the trained models RSME values are presented in Figure 16, while Table 8 contains the statistical summary of the obtained RSME values, and Figure 17 presents the amount of training iterations where the *p*-value of the *t*-test was greater than 0.05 (which indicates that the differences in results could not be significant). The best concentration (least standard deviation) of the RSME, as well as the lowest average, was observed for SVM1 model. The average RSME value of the GPR1 model was slightly worse, and the average RSMEs of ANN and ANF1 models were one order of magnitude greater than of SVM1 and GPR1 models. The RSME standard deviation of ANF1, SVM1 and GPR1 were of the same order of magnitude, while the standard deviation of the RSME of ANN was three orders of magnitude greater. Therefore, the lowest repeatability of the accuracy was observed for the ANN model, where the histogram had to be presented using logarithmic RSME scale, as the values were scattered within different orders of magnitude. The results of the *t*-tests (Figure 17) confirm that deduction, where for ANNs there was the greatest rejection rate of the null hypothesis (that the differences of performance of the trained models with the considered model are not significant).

## 4. Discussion

Three types of machine learning techniques (ANFIS, SVM, and GPR) were applied to predict nonlinear ISF of an aerostructure excited with different loads. The results were compared with each other and with ANN model obtained in previous work, for its accuracy and required computational effort, taking into account potential real-time applications in the area of operational load monitoring.

In order to find the best suited ANFIS, models with different membership function types were considered, among which, by grid search, triangular function was revealed as the type that results with the most accurate model, both for models with two and three MFs per input. The required computational effort for using the trained model for predictions grows exponentially with increasing the number of membership functions per input. Therefore, it was decided to stop at two MFs per input, as the model accuracy was considered to be sufficient, and did not improve much when introducing three MFs per input.

For SVM models, three different kernel functions were considered. For all three models grid search of hyperparameters values was performed. From the considered models, the one with gaussian kernel function (SVM1) was of accuracy by one order of magnitude higher than two others. By observing the ISF results of SVMs tests (Figure 9), it seems that SVM2 was not immune to noisy experimental input data although all the models were trained on numerical data with white gaussian noise added. This is considered to be of great importance, as in OLM one always deals with measured noisy data. SVM1 was chosen for further considerations although its computational time was higher than SVM2 and SVM3; however, the two latter did not perform well even when tested with numerical data, similar to that used in training, which is not acceptable.

Four GPR models, with different covariance functions, were considered. Their computational time and accuracy results were quite close to each other. When looking at the plots of ISF results (Figure 11) it would be hard to reveal the model with the best performance, without computing error values. Although all GPR models could be considered as acceptable, after analysis of numerical data from Table 4, GPR1 disqualified other three models, as it fitted both considered criteria (computational time and RMSE for experimental input) best.

From the abovementioned models, one of each type, considered as best, was chosen for final comparison. The accuracy of all the chosen models is acceptable and of the same order of magnitude. However, the time of computations for each model type is of different order of magnitude, ANN being the fastest, and ANFIS being the slowest. When introduced to experimentally acquired input that exceeded the training data range, only the ANN managed to return proper results outside the limits. This means that ANNs may have the strongest generalization abilities and could manage better when unusual or unpredictable event occurs.

When analyzing the statistical data of the accuracy of the trained models (study of repeatability), the ANNs fared the worst of the four considered methods. The standard deviation is of three orders of magnitude higher than for all other model types. The histogram for ANNs could not be presented with others, at it spreads on few orders of magnitude, and logarithmic scale must have been introduced for visibility. This means that training an ANN that meets the requirements for OLM is a far harder task that could require significantly more attempts than other presented methods. The other issue with ANNs it that there is no universal rule on how many neurons should be put in the hidden layer. If the number is too low, the accuracy of the model will not meet the requirements, and if the number is too high, the model could become overfitted and also of low accuracy.

Among the three other methods, SVM proved to have the lowest average value and standard deviation of RMSE from all. Moreover, its computational time is lower than ANF and GPR models. Therefore, it is recommended for OLM systems without real-time constrains, or in OLM systems where this method meets the time constraints.

For real-time systems where the computational power is not high (e.g., where the computations are performed in an embedded system with ARM architecture) or where the required time step is relatively small, one should consider two method—ANN and SVM. ANN is the fastest due to simple floating point operations in each layer; however, finding the accurate model could be hard or even impossible. In this case, SVM could be easier to implement, as it was proven to have good repeatability (by histogram and standard deviation value).

## Figures and Tables

**Table 1:** Considered prediction models.

Model Designation	Description
ANF1	ANFIS with two MFs per input
ANF2	ANFIS with three MFs per input
SVM1	SVM with gaussian kernel function
SVM2	SVM with linear kernel function
SVM3	SVM with polynomial kernel function
GPR1	GPR with squared exponential covariance function
GPR2	GPR with exponential covariance function
GPR3	GPR with Matern 5/2 covariance function
GPR4	GPR with rational quadratic covariance function

**Table 2:** Comparison of accuracy and computational time for obtained ANFIS models.

Model	Computational Time [ms]	RMSE (Numerical Input)	RMSE (Experimental Input)
ANF1	144.0	0.0048	0.0093
ANF2	1539.3	0.0056	0.0084

**Table 3:** Comparison of accuracy and computational time for obtained SVM models.

Model	Computational Time [ms]	RMSE (Numerical Input)	RMSE (Experimental Input)
SVM1	3.9	0.0022	0.0023
SVM2	2.3	0.0198	0.0187
SVM3	2.0	0.0147	0.0165

**Table 4:** Comparison of accuracy and computational time for obtained GPR models.

Model	Computational Time [ms]	RMSE (Numerical Input)	RMSE (Experimental Input)
GPR1	16.8	0.0024	0.0025
GPR2	19.0	0.0011	0.0036
GPR3	24.2	0.0017	0.0032
GPR4	29.9	0.0018	0.0034

**Table 5:** Comparison of accuracy and computational time for chosen models.

Model	Computational Time [ms]	RMSE (Numerical Input)	RMSE (Experimental Input)
ANF1	144.0	0.0048	0.0093
SVM1	2.4	0.0022	0.0023
GPR1	20.7	0.0024	0.0025
ANN	0.7	0.0020	0.0074

**Table 6:** Resultant *p*-values of comparing different models with *t*-test (numerical input).

Model	ANF1	SVM1	GPR1	ANN
**ANF1**	X	0.0453	0.0365	0.0062
**SVM1**	0.0453	X	0.3818	0.0148
**GPR1**	0.0365	0.3818	X	0.0696
**ANN**	0.0062	0.0148	0.0696	X

**Table 7:** Resultant *p*-values of comparing different models with *t*-test (experimental input).

Model	ANF1	SVM1	GPR1	ANN
**ANF1**	X	0.3182	0.6637	0.0053
**SVM1**	0.3182	X	0.0217	0.0001
**GPR1**	0.6637	0.0217	X	0.0000
**ANN**	0.0053	0.0001	0.0000	X

**Table 8:** Comparison of statistical RMSE data for training 1000 models.

Model	Minimum Value	Maximum Value	Average Value	Standard Deviation
ANF1	0.0090	0.0121	0.0107	4.98 × 10^−4^
SVM1	0.0023	0.0038	0.0028	2.17 × 10^−4^
GPR1	0.0028	0.0066	0.0040	5.06 × 10^−4^
ANN	0.0050	4.9595	0.0893	0.2452

## Supplementary Materials

The following are available online at https://www.mdpi.com/1424-8220/20/24/7087/s1, Spreadsheet S1: Training data with white gaussian noise added to inputs; Matlab Files of the obtained prediction models—File F1: ANF1, File F2: ANF2, File F3: SVM1, File F4: SVM2, File F5: SVM3, File F6: GPR1, File F7: GPR2, File F8: GPR3, File F9: GPR4.

## References

[1] Aldridge N., Foote P., Read I. (2000). Operational Load Monitoring for Aircraft & Maritime Applications. Strain.

[2] Guo H., Xiao G., Mrad N., Yao J. (2013). Echelle Diffractive Grating Based Wavelength Interrogator for Potential Aerospace Applications. J. Light. Technol..

[3] Carden E.P., Fanning P. (2004). Vibration Based Condition Monitoring: A Review. Struct. Health Monit..

[4] Kurnyta A., Zielinski W., Reymer P., Dziendzikowski M. Operational Load Monitoring System Implementation for Su-22UM3K Aging Aircraft. Proceedings of the Structural Health Monitoring 2017 Real-Time Material State Awareness and Data-Driven Safety Assurance Proceedings of the Eleventh International Workshop on Structural Health Monitoring.

[5] Giurgiutiu V. (2016). Chapter 10-SHM of Fatigue Degradation and Other In-Service Damage of Aerospace Composites. Structural Health Monitoring of Aerospace Composites.

[6] Willis S., Bos M.J. (2009). Olm: A Hands-on Approach. ICAF 2009, Bridging the Gap between Theory and Operational Practice.

[7] Mitra M., Gopalakrishnan S. (2016). Guided wave based structural health monitoring: A review. Smart Mater. Struct..

[8] Fan W., Qiao P. (2010). Vibration-based Damage Identification Methods: A Review and Comparative Study. Struct. Health Monit..

[9] Davoudi R., Miller G., Kutz J.N. (2018). Data-driven vision-based inspection for reinforced concrete beams and slabs: Quantitative damage and load estimation. Autom. Constr..

[10] Boller C., Staszewski W.J. (2004). Aircraft Structural Health and Usage Monitoring. Health Monitoring of Aerospace Structures.

[11] In Flight Load Determination in Critical Structure Elements Based on Operational Load Monitoring System. https://www.ndt.net/article/ewshm2018/papers/0279-Kurnyta.pdf.

[12] Lecler S., Meyrueis P., Yasin M., Harun S.W., Arof H. (2012). Intrinsic Optical Fiber Sensor. Fiber Optic Sensors.

[13] Betz D.C., Staszewski W.J., Thursby G., Culshaw B. (2006). Multi-functional fibre Bragg grating sensors for fatigue crack detection in metallic structures. Proc. Inst. Mech. Eng. Part G J. Aerosp. Eng..

[14] Guo H., Xiao G., Mrad N., Yao J. (2011). Fiber Optic Sensors for Structural Health Monitoring of Air Platforms. Sensors.

[15] Mucha W., Kuś W., Viana J.C., Nunes J. (2020). Operational Load Monitoring of a Composite Panel Using Artificial Neural Networks. Sensors.

[16] Raj S., Praveen Chand G.S.S., Ray K.C. (2015). ARM-based arrhythmia beat monitoring system. Microprocess. Microsyst..

[17] Silva L.C., Filho E.F.S., Albuquerque M.C., Silva I.C., Farias C.T. (2020). Embedded decision support system for ultrasound nondestructive evaluation based on extreme learning machines. Comput. Electr. Eng..

[18] Civera J.I., Garcia-Brejio E., Miró N.L., Gil Sánchez L., Baixauli J.G., Gil I.R., Peris R.M., Fillol M.A. (2011). Artificial neural network onto eight bit microcontroller for Secchi depth calculation. Sens. Actuators B Chem..

[19] Bayindir R., Sagiroglu S., Colak I. (2009). An intelligent power factor corrector for power system using artificial neural networks. Electr. Power Syst. Res..

[20] Mucha W. (2019). Application of Artificial Neural Networks in Hybrid Simulation. Appl. Sci..

[21] Zhang C., Zhao J., Li F., Jia H., Tian J. (2011). Support Vector Machine Algorithm for Real-Time Detection of VF Signals. Procedia Environ. Sci..

[22] Arsalane A., El Barbri N., Tabyaoui A., Arsalane A., Rhofir K., Halimi A. (2018). An embedded system based on DSP platform and PCA-SVM algorithms for rapid beef meat freshness prediction and identification. Comput. Electron. Agric..

[23] Dey S., Kedia M., Agarwal N., Basu A. Embedded Support Vector Machine: Architectural Enhancements and Evaluation. Proceedings of the 20th International Conference on VLSI Design held jointly with 6th International Conference on Embedded Systems (VLSID’07).

[24] Batista G.C., Oliveira D.L., Saotome O., Silva W.S. (2020). A low-power asynchronous hardware implementation of a novel SVM classifier, with an application in a speech recognition system. Microelectron. J..

[25] Afifi S., GholamHosseini H., Sinha R. (2019). A system on chip for melanoma detection using FPGA-based SVM classifier. Microprocess. Microsyst..

[26] Torres-Salomao L.A., Anzurez-Marin J., Orozco-Sixtos J.M., Ramirez-Zavala S. ANFIS data driven modeling and real-time Fuzzy Logic Controller test for a Two Tanks Hydraulic System. Proceedings of the 2015 IEEE International Conference on Evolving and Adaptive Intelligent Systems (EAIS).

[27] Hari V.M., Lakshmi P., Kalaivani R. Design and implementation of Adaptive Neuro Fuzzy Inference System for an experimental Active Suspension System. Proceedings of the 2015 International Conference on Robotics, Automation, Control and Embedded Systems (RACE).

[28] Armendariz J., Parra-Vega V., García-Rodríguez R., Rosales S. (2014). Neuro-fuzzy self-tuning of PID control for semiglobal exponential tracking of robot arms. Appl. Soft Comput..

[29] Pothal J.K., Parhi D.R. (2015). Navigation of multiple mobile robots in a highly clutter terrains using adaptive neuro-fuzzy inference system. Robot. Auton. Syst..

[30] Karakuzu C., Karakaya F., Çavuşlu M.A. (2016). FPGA implementation of neuro-fuzzy system with improved PSO learning. Neural Netw..

[31] Maldonado Y., Castillo O., Melin P. (2014). A multi-objective optimization of type-2 fuzzy control speed in FPGAs. Appl. Soft Comput..

[32] Danzomo B.A., Salami M.J.E., Khan R. Hardware Implementation of ANFIS Controller for Gas-Particle Separations in Wet Scrubber System. Proceedings of the 2014 International Conference on Computer and Communication Engineering.

[33] Sakib S.N., Ane T., Matin N., Kaiser M.S. An intelligent flood monitoring system for Bangladesh using wireless sensor network. Proceedings of the 2016 5th International Conference on Informatics, Electronics and Vision (ICIEV).

[34] Mishra R.N., Mohanty K.B. (2016). Real time implementation of an ANFIS-based induction motor drive via feedback linearization for performance enhancement. Eng. Sci. Technol. Int. J..

[35] Zanella L., Porru M., Bottegal G., Gallucci F., van Sint Annaland M., Özkan L., Kiss A.A., Zondervan E., Lakerveld R., Özkan L. (2019). Real-time determination of optimal switching times for a H_2_ production process with CO_2_ capture using Gaussian Process Regression models. Computer Aided Chemical Engineering.

[36] Gijsberts A., Metta G. (2013). Real-time model learning using Incremental Sparse Spectrum Gaussian Process Regression. Neural Netw..

[37] Tsai S.W., Wu E.M. (1971). A General Theory of Strength for Anisotropic Materials. J. Compos. Mater..

[38] Jang J.-S.R. (1993). ANFIS: Adaptive-network-based fuzzy inference system. IEEE Trans. Syst. Man Cybern..

[39] Takagi T., Sugeno M. (1985). Fuzzy identification of systems and its applications to modeling and control. IEEE Trans. Syst. Man Cybern..

[40] Celikyilmaz A., Türkşen I.B. (2009). Modeling Uncertainty with Fuzzy Logic. Towards New Evol. Comput..

[41] Mathur N., Glesk I., Buis A. (2016). Comparison of adaptive neuro-fuzzy inference system (ANFIS) and Gaussian processes for machine learning (GPML) algorithms for the prediction of skin temperature in lower limb prostheses. Med. Eng. Phys..

[42] Cabalar A.F., Cevik A., Gokceoglu C. (2012). Some applications of Adaptive Neuro-Fuzzy Inference System (ANFIS) in geotechnical engineering. Comput. Geotech..

[43] Naresh C., Bose P.S.C., Rao C.S., Selvaraj N. (2020). Prediction of cutting force of AISI 304 stainless steel during laser-assisted turning process using ANFIS. Mater. Today Proc..

[44] Zhuang X., Yu T., Sun Z., Song K. (2020). Wear prediction of a mechanism with multiple joints based on ANFIS. Eng. Fail. Anal..

[45] Kar S., Pandit A.R., Biswal K. (2020). Prediction of FRP shear contribution for wrapped shear deficient RC beams using adaptive neuro-fuzzy inference system (ANFIS). Structures.

[46] Neuro-Adaptive Learning and ANFIS-MATLAB & Simulink. https://www.mathworks.com/help/fuzzy/neuro-adaptive-learning-and-anfis.html.

[47] Vapnik V. (2013). The Nature of Statistical Learning Theory.

[48] Aizerman M.A. (1964). Theoretical foundations of the potential function method in pattern recognition learning. Autom. Remote Control.

[49] Smola A.J., Schölkopf B. (2004). A tutorial on support vector regression. Stat. Comput..

[50] Gopalakrishnan K., Kim S. (2011). Support Vector Machines Approach to HMA Stiffness Prediction. J. Eng. Mech..

[51] Zhou J., Li X. (2011). Evaluating the Thickness of Broken Rock Zone for Deep Roadways using Nonlinear SVMs and Multiple Linear Regression Model. Procedia Eng..

[52] Zhang H.-T., Gao M.-X. (2018). The Application of Support Vector Machine (SVM) Regression Method in Tunnel Fires. Procedia Eng..

[53] Tuttle J.F., Blackburn L.D., Powell K. (2020). On-line classification of coal combustion quality using nonlinear SVM for improved neural network NOx emission rate prediction. Comput. Chem. Eng..

[54] Statistics and Machine Learning Toolbox. https://www.mathworks.com/products/statistics.html.

[55] MacKay D.J., Mac Kay D.J. (2003). Information Theory, Inference and Learning Algorithms.

[56] Jin Z.-J., Qian H., Zhu M.-L. Gaussian processes in inverse reinforcement learning. Proceedings of the 2010 International Conference on Machine Learning and Cybernetics.

[57] Xiao F., Li C., Fan Y., Yang G., Tang X. (2021). State of charge estimation for lithium-ion battery based on Gaussian process regression with deep recurrent kernel. Int. J. Electr. Power Energy Syst..

[58] Pensoneault A., Yang X., Zhu X. (2020). Nonnegativity-enforced Gaussian process regression. Theor. Appl. Mech. Lett..

[59] Baiz A.A., Ahmadi H., Shariatmadari F., Torshizi M.A.K. (2020). A Gaussian process regression model to predict energy contents of corn for poultry. Poult. Sci..

[60] Tonner P.D., Darnell C.L., Engelhardt B.E., Schmid A.K. (2017). Detecting differential growth of microbial populations with Gaussian process regression. Genome Res..

[61] Roberts S., Osborne M., Ebden M., Reece S., Gibson N., Aigrain S. (2013). Gaussian processes for time-series modelling. Philos. Trans. R. Soc. A Math. Phys. Eng. Sci..

[62] Swain P.S., Stevenson K., Leary A., Montano-Gutierrez L.F., Clark I.B., Vogel J., Pilizota T. (2016). Inferring time derivatives including cell growth rates using Gaussian processes. Nat. Commun..

[63] Li L.-L., Zhang X.-B., Tseng M.-L., Zhou Y. (2019). Optimal scale Gaussian process regression model in Insulated Gate Bipolar Transistor remaining life prediction. Appl. Soft Comput..

[64] Huttunen J.M.J., Kärkkäinen L., Lindholm H. (2019). Pulse transit time estimation of aortic pulse wave velocity and blood pressure using machine learning and simulated training data. PLoS Comput. Biol..

[65] Richardson R.R., Osborne M., Howey D.A. (2017). Gaussian process regression for forecasting battery state of health. J. Power Sources.

[66] Rasmussen C.E., Williams C. (2006). Gaussian Processes for Machine Learning.

